# Draft genome sequence of a non-tuberculous Mycobacterium strain isolated from a clinical urine sample

**DOI:** 10.1099/acmi.0.001110.v3

**Published:** 2026-01-28

**Authors:** Joanna E. Rivas Ramos, Paul R. Johnston, Robert J. H. Hammond, Matthew T. G. Holden, Derek J. Sloan, Andreas F. Haag

**Affiliations:** 1 School of Medicine, University of St Andrews, St Andrews, Scotland, UK

**Keywords:** antimicrobial susceptibility, draft genome sequence, *Mycobacterium*, non-tuberculous mycobacteria (NTM), phylogenetic analysis

## Abstract

Here, we report the draft sequence of a rapid-growing nontuberculous *Mycobacterium* isolated from a urine sample at the Scottish Mycobacteria Reference Laboratory, Royal Infirmary of Edinburgh, UK. The reported genome has a length of 6,749,454 bp, a G+C content of 67.2 mol% and 6,336 protein CDSs. Average nucleotide identity (ANI) analysis identified *Mycobacterium vanbaalenii* PYR-1 as the closest relative (83.32% ANI), indicating that this isolate likely represents a novel species within the genus. Notably, phenotypic characterization revealed a distinct antimicrobial resistance (AMR) profile. This assembly provides a valuable resource for studying the evolution of AMR mechanisms in nontuberculous mycobacteria and offers insight into resistance phenotypes observed in clinical isolates.

## Data Summary

All data are available for *Mycobacterium* spp. strain JER01, which has been deposited in the European Nucleotide Archive under BioProject accession number PRJNA1229527, and the accession for the assembly is GCA_965295185.1. The BioSample accession number is SAMN47159797. The raw Oxford Nanopore and the Illumina read data can be found under run accessions SRR32527371–SRR32527375.

## Introduction

The genus *Mycobacterium*, within the phylum *Actinobacteria*, comprises over 190 named species, including major human pathogens such as *Mycobacterium tuberculosis* and *Mycobacterium leprae*, the causative agents of tuberculosis and leprosy, respectively [1]. In addition to these, the genus includes numerous non-tuberculous mycobacteria (NTMs), which are increasingly recognized as opportunistic pathogens, particularly in individuals with underlying lung disease or immunosuppression. Clinically relevant NTMs include *Mycobacterium abscessus* and *Mycobacterium avium*. However, many NTMs are primarily environmental organisms with limited or no pathogenic potential [2].

The taxonomic diversity and variable pathogenic potential of NTMs underscore the importance of continued characterization of newly isolated strains. Here, we report the genome sequence and preliminary characterization of a previously unclassified mycobacterial isolate, designated JER01, obtained from a clinical specimen in Scotland. Whole-genome sequencing was undertaken to establish its taxonomic placement, assess its potential as a model organism for drug resistance studies and provide insights into the genetic diversity of the *Mycobacterium* genus.

## Results and discussion

**Fig. 1.**
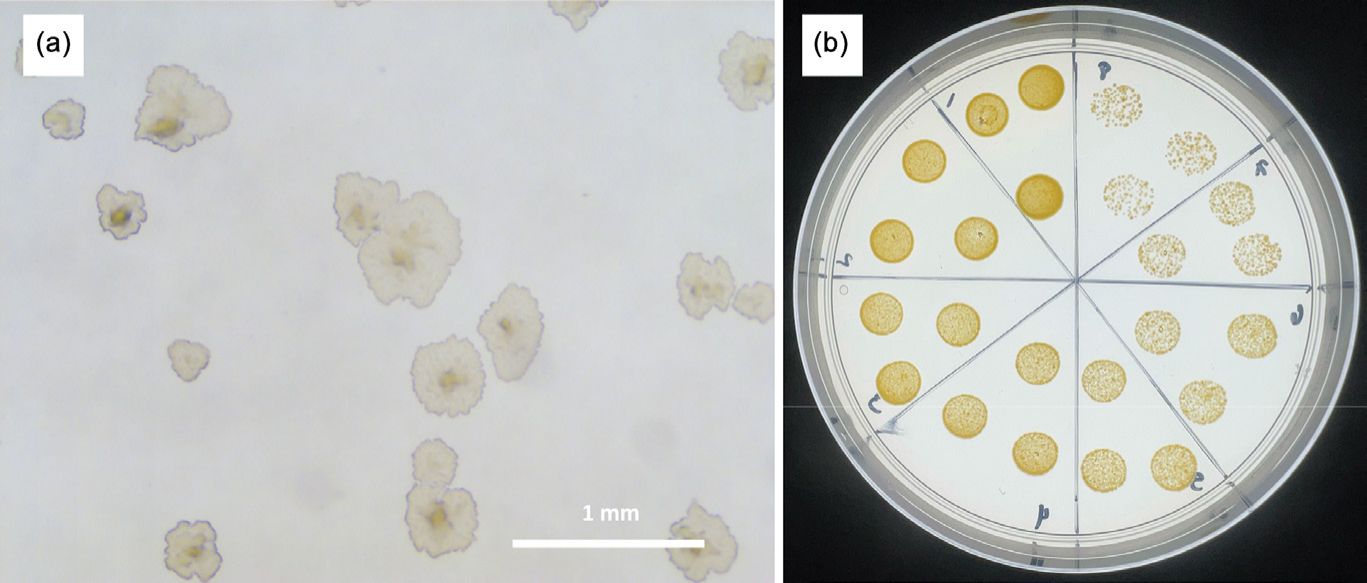
General phenotypic characteristics of *Mycobacterium* spp. JER01. (**a**) Representative example of JER01 presenting a rough morphology after 5 days of incubation at 30 °C on Middlebrook 7H10 agar plate. (**b**) JER01 displays a bright yellow scotochromogenic colony after 5 days of incubation at 30 °C on Middlebrook 7H10 agar. Three 10 µl aliquots per dilution of a tenfold serial dilution series (10^−1^ to 10^−8^) of JER01 in PBS were spotted onto a Middlebrook 7H10 agar plate and incubated for 5 days at 30 °C.

JER01 was recovered in 2010 from a male urine sample submitted to the Scottish Mycobacteria Reference Laboratory, Royal Infirmary of Edinburgh, UK. Preliminary *hsp65* gene sequencing confirmed its identity as a non-tuberculous *Mycobacterium*. JER01 was cultured on Middlebrook 7H10 agar and exhibited robust growth at 30 and 37 °C, but no growth at 42 °C. Colonies became visible after 4 days of incubation, consistent with a rapid-growing mycobacterial phenotype. The colonies were round with rough edges, dry in appearance and yellow-orange in colour (Fig. 1a, b, respectively). Pigmentation was observed under all conditions, independent of light exposure or incubation temperature, indicating that the organism is scotochromogenic [3].

Antimicrobial susceptibility testing was conducted using the microdilution broth method [4], with results interpreted using the European Committee on Antimicrobial Susceptibility Testing (EUCAST) and the Clinical and Laboratory Standards Institute (CLSI) clinical breakpoints established for *M. tuberculosis* H37Rv [4,7]. JER01 was susceptible to rifampicin, ethambutol, moxifloxacin, levofloxacin, bedaquiline and linezolid, but resistant to isoniazid, pyrazinamide and pretomanid (Table 1).

**Table 1.** Antimicrobial susceptibility testing results for JER01 isolate using the microdilution broth method and interpretation

Antimicrobial agent	MIC (µg ml^−1^)
Susceptible (S)*	
Rifampicin	0.002
Ethambutol	0.5
Moxifloxacin	0.0625
Levofloxacin	0.125
Bedaquiline	0.125
Linezolid	0.5
Resistant (R)*	
Isoniazid	32
Pyrazinamide	>512
Pretomanid	>512

*Susceptibility based on the clinical breakpoints and critical concentrations used as references determined using the Mycobacterial Growth Indicator Tube according to CLSI and EUCAST.

To establish the taxonomic identity of this potentially novel isolate and assess its suitability as a model organism for drug resistance studies, whole-genome sequencing using Oxford Nanopore Technologies (ONT) and polished by using Illumina short reads was performed. JER01 was cultured in Middlebrook 7H9 broth until it reached an OD₆₀₀ of 2.0 (~10^9^ c.f.u.), next bacteria were pelleted (4,000 ***g*** for 10 min), resuspended in a cryopreservative (Microbank; Pro-Lab Diagnostics UK, UK) and shipped to MicrobesNG (Birmingham, UK) for sequencing. DNA extraction and processing were conducted following MicrobesNG (Birmingham, UK) in-house protocols. For ONT sequencing, libraries were prepared using the ONT SQK-RBK114.96 rapid barcoding kit (ONT, UK) using 200–400 ng of high-molecular-weight DNA and sequenced on a GridION platform (ONT, UK) using an R10.4.1 flow cell, with the r1041_e82_400bps_hac_v4.2.0 model. Reads were randomly subsampled to 50X coverage using Rasusa v0.7.1 [8]. Illumina short-read sequencing was performed using the Nextera XT Library Prep Kit (Illumina, San Diego, USA) following the manufacturer’s protocol with the following modifications: input DNA was increased 2-fold, and PCR elongation time was increased to 45 s. Libraries were sequenced on an Illumina NovaSeq 6000 (Illumina, San Diego, USA) using a 250 bp paired-end protocol. Long and short reads were assembled using Unicycler (v0.5.1) [9], and assembly quality was assessed with QUAST (v5.3.0) [10] and visualized using Bandage (v0.9.0) [11].

The draft genome of JER01 comprised 6,749,454 bp with a G+C content of 67.2 mol% and an N50 of 5,968,660 bp. The assembly yielded 5 contigs: a circularized chromosome of 5,968,660 bp, 2 linear contigs (261,025 and 219,653 bp) and 2 smaller circular contigs (190,769 and 109,347 bp, respectively). The circular nature of the smaller contigs suggests the presence of plasmids [12]. Genome annotation using Bakta version 1.11.0 [13,15] identified 6,336 CDSs, 6 rRNAs, 2 tmRNAs and 49 tRNAs. To determine the taxonomic placement of JER01, we compared the genome to 20 well-characterized *Mycobacterium* species using the OrthoANI algorithm via the EzBioCloud platform (available at https://www.ezbiocloud.net/tools/orthoani) [16], Artemis Comparison Tool (ACT) v18.2.0 [17] and the pangenome analysis tool Panaroo v1.4.0 [18]. The closest relative was *Mycobacterium vanbaalenii* PYR-1, with an average nucleotide identity of 83.32%. A maximum likelihood phylogeny was constructed based on core proteins identified by Panaroo (minimum 50% sequence identity and coverage, present in ≥80% of genomes). The tree was generated with IQ-TREE v3.0.1 [19] using 1,000 ultrafast bootstraps and visualized with the Interactive Tree of Life (iTOL) v5 server [20].

**Fig. 2.**
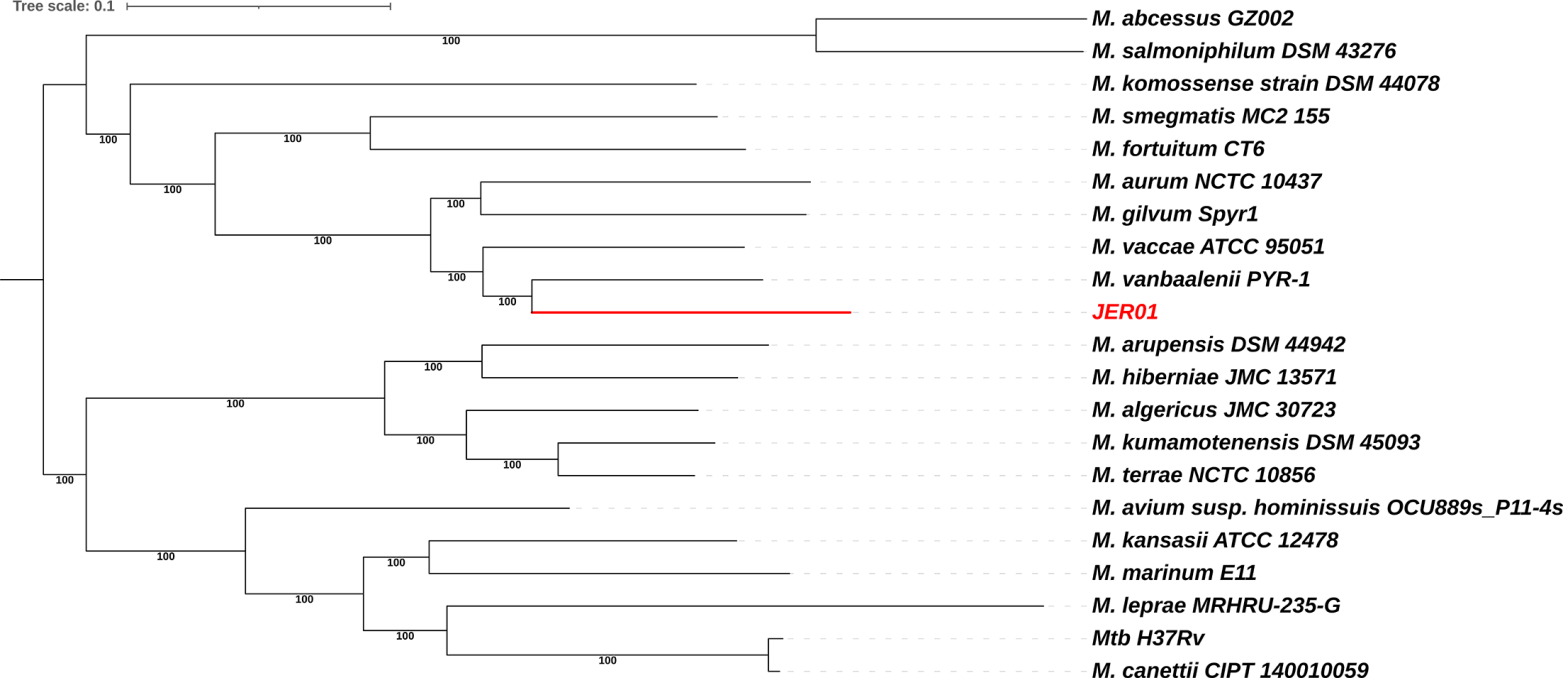
Core genome phylogeny of representative *Mycobacterium* species. A phylogenetic tree was created using core genes detected by Panaroo of representative species of each mycobacterial subgenus (*n*=20), where the red node and red text indicate the location of the JER01 isolate presented. Panaroo used clustal for the alignments of the different genes using only the core genes, and IQ-TREE was used to calculate the phylogenetic tree with 1,000 ultrafast bootstraps. *M. abscessus* GZ002 was used as an outgroup to root the phylogeny. The tree was edited using iTOL v5. The following genomes served as sources for the pangenome analysis used in the alignment and tree: *Mycobacterium marinum* E11 (GenBank accession number NZ_HG917972.2), *M. avium subspecies hominissuis* OCU889s_P11-4s (GenBank accession number NZ_CP018019.1), *Mycobacterium canetti* CIPT 140010059 (GenBank accession number NC_015848.1), *M. leprae* MRHRU-235-G (GenBank accession number NZ_CP029543.1), *Mycobacterium kansasii* ATCC 12478 (GenBank accession number NC_022663.1), *M. tuberculosis* H37Rv (GenBank accession number AL123456.3), *Mycobacterium smegmatis* MC2 155 (GenBank accession number NZ_CP009494), *Mycobacterium aurum* NCTC10437 (GenBank accession number NZ_LR134356), *Mycobacterium fortuitum* CT6 (GenBank accession number CP011269.1), *M. vaccae* ATCC 95051 (GenBank accession number NZ_CP011491), *Mycobacterium Spyr1* (GenBank accession number NC_014814), *M. vanbaalenii* PYR-1 (GenBank accession number NC_008726), *Mycobacterium komossense* strain DSM 44078 (GenBank accession number NZ_JACKTY010000001), *Mycobacterium algericus* strain JCM 30723 (GenBank accession number NZ_BLKY01000001.1), *Mycobacterium hiberniae* JCM 13571 (GenBank accession number NZ_AP022609), *Mycobacterium kumamotonensis* strain DSM 45093 (GenBank accession number NZ_MVHU01000100.1), *Mycobacterium terrae* NCTC10856 (GenBank accession number NZ_LT906469.1), *Mycobacterium arupensis* strain DSM 44942 (GenBank accession number NZ_MVHH01000001.1), *Mycobacterium salmoniphilum* strain DSM 43276 (GenBank accession number NZ_CP024633.1) and *M. abscessus* strain GZ002 (GenBank accession number NZ_CP034181.1).

Phylogenetic analysis positioned JER01 within the Fortuitum–Vaccae clade of fast-growing mycobacteria, clustering closely with *M. vanbaalenii* PYR-1 and *Mycobacterium vaccae* ATCC 95051 (Fig. 2). In contrast, slow-growing species such as *M. tuberculosis* H37Rv and *M. leprae* MRHRU-235-G clustered within a distant clade encompassing members of the Tuberculosis–Simiae, Abscessus–Chelonae and Terrae groups.

These results support the designation of JER01 as a likely novel species within the Fortuitum–Vaccae clade. The combination of its distinct taxonomic placement and unique antimicrobial resistance profile, including resistance to key anti-tuberculosis drugs like isoniazid, pyrazinamide and pretomanid, while remaining susceptible to key antibiotics such as rifampicin, bedaquiline and linezolid, suggests that JER01 could be a valuable reference for future comparative studies of mycobacterial evolution and drug resistance mechanisms. Its non-pathogenic nature and specific resistance pattern offer a unique opportunity to explore the genetic basis of drug resistance in a biosafety level 1 setting, with potential relevance for understanding resistance evolution in *Mtb*.

## Abbreviations

AMR: antimicrobial resistance
ANI: average nucleotide identity
CLSI: Clinical and Laboratory Standards Institute
EUCAST: European Committee on Antimicrobial Susceptibility Testing
iTOL: Interactive Tree of Life
NTM: non-tuberculous mycobacteria
ONT: Oxford Nanopore Technologies

## References

[1] Gupta RS, Lo B, Son J (2018). Phylogenomics and comparative genomic studies robustly support division of the genus *Mycobacterium* into an emended genus *Mycobacterium* and four novel genera. Front Microbiol.

[2] Meehan CJ, Barco RA, Loh Y-HE, Cogneau S, Rigouts L (2021). Reconstituting the genus *Mycobacterium*. Int J Syst Evol Microbiol.

[3] Maboni G, Prakash N, Moreira MAS (2024). Review of methods for detection and characterization of non-tuberculous mycobacteria in aquatic organisms. J Vet Diagn Invest.

[4] Schön T, Werngren J, Machado D, Borroni E, Wijkander M (2020). Antimicrobial susceptibility testing of *Mycobacterium tuberculosis* complex isolates - the EUCAST broth microdilution reference method for MIC determination. Clin Microbiol Infect.

[5] European Committee on Antimicrobial Susceptibility Testing (2025). Breakpoint tables for interpretation of mics and zone diameters. Version 15.0.

[6] European Committee on Antimicrobial Susceptibility Testing (AMST subcommittee) (2019). Reference protocol for MIC determination of anti-tuberculous agents against isolates of the Mycobacterium tuberculosis complex in Middlebrook 7H9 broth.

[7] Brown-Elliott BA, Woods GL (2019). Antimycobacterial susceptibility testing of nontuberculous mycobacteria. J Clin Microbiol.

[8] Hall M (2022). Rasusa: randomly subsample sequencing reads to a specified coverage. JOSS.

[9] Wick RR, Judd LM, Gorrie CL, Holt KE (2017). Unicycler: resolving bacterial genome assemblies from short and long sequencing reads. PLoS Comput Biol.

[10] Gurevich A, Saveliev V, Vyahhi N, Tesler G (2013). QUAST: quality assessment tool for genome assemblies. Bioinformatics.

[11] Wick RR, Schultz MB, Zobel J, Holt KE (2015). Bandage: interactive visualization of de novo genome assemblies. Bioinformatics.

[12] Hendrix J, Epperson LE, Durbin D, Honda JR, Strong M (2021). Intraspecies plasmid and genomic variation of *Mycobacterium kubicae* revealed by the complete genome sequences of two clinical isolates. Microb Genom.

[13] Tatusova T, DiCuccio M, Badretdin A, Chetvernin V, Nawrocki EP (2016). NCBI prokaryotic genome annotation pipeline. Nucleic Acids Res.

[14] Haft DH, DiCuccio M, Badretdin A, Brover V, Chetvernin V (2018). RefSeq: an update on prokaryotic genome annotation and curation. Nucleic Acids Res.

[15] Li W, O’Neill KR, Haft DH, DiCuccio M, Chetvernin V (2021). RefSeq: expanding the prokaryotic genome annotation pipeline reach with protein family model curation. Nucleic Acids Res.

[16] Lee I, Ouk Kim Y, Park SC, Chun J (2016). OrthoANI: an improved algorithm and software for calculating average nucleotide identity. Int J Syst Evol Microbiol.

[17] Carver TJ, Rutherford KM, Berriman M, Rajandream M-A, Barrell BG (2005). ACT: the Artemis Comparison Tool. Bioinformatics.

[18] Tonkin-Hill G, MacAlasdair N, Ruis C, Weimann A, Horesh G (2020). Producing polished prokaryotic pangenomes with the Panaroo pipeline. Genome Biol.

[19] Nguyen L-T, Schmidt HA, von Haeseler A, Minh BQ (2015). IQ-TREE: a fast and effective stochastic algorithm for estimating maximum-likelihood phylogenies. Mol Biol Evol.

[20] Letunic I, Bork P (2021). Interactive Tree Of Life (iTOL) v5: an online tool for phylogenetic tree display and annotation. Nucleic Acids Res.

[21] Percival‐Alwyn L, Barnes I, Clark MD, Cockram J, Coffey MP (2025). UKCropDiversity‐HPC: a collaborative high‐performance computing resource approach for sustainable agriculture and biodiversity conservation. *Plants People Planet*.

